# Employee Wellbeing: Evaluating a Wellbeing Intervention in Two Settings

**DOI:** 10.3389/fpsyg.2017.00505

**Published:** 2017-04-04

**Authors:** Alexis Keeman, Katharina Näswall, Sanna Malinen, Joana Kuntz

**Affiliations:** Department of Psychology, University of CanterburyChristchurch, New Zealand

**Keywords:** the Wellbeing Game, the Five Ways to Wellbeing, changes in wellbeing, longitudinal studies, experimental studies

## Abstract

This research presents two studies conducted to evaluate the Wellbeing Game in two different contexts: In a student sample and in an organizational setting. Study 1 investigated the efficacy of the Wellbeing Game, in terms of its effect of wellbeing, stress, and an image valence test, among 60 university students. The results showed that after playing the Wellbeing Game, students reported a significant positive change in wellbeing compared to those who did not play the Wellbeing Game, but there was no decrease in stress or any change in classification of image valence. Study 2 evaluated the Wellbeing Game in an organizational context. Employees (*n* = 52) in a financial organization played the Wellbeing Game for 4 weeks and answered survey questions about wellbeing and stress at the beginning and end of this period. The results showed that after playing the Wellbeing Game, employees reported lower stress levels, and higher wellbeing levels for those who felt that it had helped them connect more with colleagues. The results from the two studies provide preliminary support that the Wellbeing Game may be an effective wellbeing intervention tool in both an organization and a non-organizational context.

## Introduction

The developed world is now, to a large extent, driven by the knowledge economy, where an increasing number of jobs focus on technology and information production requiring employees with specialized skillsets, making individuals difficult to replace (Hellgren et al., 2008). Supporting and retaining employees is therefore important for organizational success.

The typical person spends one quarter of their adult life at work, and for many work a key life pursuit. Feeling good and functioning well at work are therefore key components of a person's overall wellbeing. Experiencing a high level of wellbeing is associated with a range of positive organizational attitudes. These include superior work performance (Lyubomirsky et al., 2005), low turnover intentions, low actual turnover (Boehm and Lyubomirsky, 2008), greater effort and thought put into work, less absenteeism and fewer work related injuries (Keyes and Grzywacz, 2005). Given that work affects wellbeing, and that wellbeing is important for organizational success, it is in an organization's best interests to support and promote wellbeing at work (Dewe and Cooper, 2012; Hone et al., 2015).

Despite the many positive organizational attitudes associated with employee wellbeing, organizations have traditionally focused on reducing employee stress rather than increasing employee wellbeing (Hone et al., 2015). Stress occurs when there is a perceived imbalance between personal resources and perceived demands in a given situation (Lazarus and Folkman, 1984). Stress is linked to decreased productivity and therefore reduced profit (Ford et al., 2011), prompting organizations to focus on implementing stress reduction interventions (Kelloway and Day, 2005).

Traditional workplace wellbeing interventions have focused on individual stress management, but evidence suggests that traditional stress interventions are often ineffective in the long term and do not result in improved organizational outcomes (LaMontagne et al., 2007; Baumeister and Alghamdi, 2015; Vanhove et al., 2016). Stress and wellbeing constitute separate, but related, constructs. Just as the absence of mental illness does not equate mental health (Keyes, 2005), the absence of stress does not equate wellbeing. However, evidence suggests that flourishing may provide a buffer against the negative effects of stress (Keyes and Grzywacz, 2005), indicating that investment in promotion of wellbeing may contribute to stress reduction while also producing additional benefits associated with flourishing at work (Hone et al., 2015).

One effective way to increase wellbeing is to incorporate five broad categories of positive activities in our day-to-day lives (Aked et al., 2009), which have been empirically related to flourishing (Hone et al., 2015). These five categories, named the Five Ways to Wellbeing have been labeled *Connect, Be Active, Take Notice, Keep Learning*, and *Give*. To encourage their adoption, The Five Ways have been incorporated in an online tool: The Wellbeing Game. The present study investigates whether engaging with the Wellbeing Game is related to higher levels of wellbeing and a change in the perception of positive stimuli, and explores whether the Wellbeing Game can be used as an organizational tool to support higher wellbeing and lower stress, as well as the improvement of organizational attitudes.

## Wellbeing at work

Wellbeing has been defined according to two main, but distinct, perspectives. According to the hedonic perspective, wellbeing is described as happiness (Ryan and Deci, 2001). This perspective emphasizes the importance of three components: Life satisfaction, the presence of positive mood, and the absence of negative mood (Diener et al., 1998).

In the second, eudaimonic, perspective wellbeing is described in terms of self-actualization, proposing that true happiness is found in expressing virtue (Dewe and Cooper, 2012). The eudaimonic perspective views engagement in activities which foster human growth, such as autonomy, personal growth, self-acceptance, life purpose, mastery, and positive relatedness, as essential to wellbeing (Ryff and Keyes, 1995).

The present study draws on research which proposes that a combination of both perspectives is more accurate, as wellbeing can be viewed as a multidimensional phenomenon that encompasses both eudaimonic and hedonic aspects (Fisher, 2014). In the present study, wellbeing is conceptualized as the combination of feeling good (hedonism) and functioning well (eudemonia) (Aked et al., 2009).

Wellbeing at work refers to a subjective perception of general satisfaction with and positive feelings toward work. It has also been suggested that conceptualizations of wellbeing at work (and in general) should include a component of social relationships, as this is a key component of an individual's positive experiences at work (Fisher, 2014). Research shows that employees with a high level of wellbeing put greater thought and effort into their work (Canaff and Wright, 2004; Keyes and Grzywacz, 2005; Day and Randell, 2014). Poor psychological health, such as depressed mood, anxiety and fatigue, are related to decrements in cognitive resources, and increased focus on negative or irrelevant information (Ford et al., 2011), which in turn is related to poorer performance (Taris, 2006). Poor psychological health is also related less energy and motivation to engage in positive behaviors at work, resulting in poorer contextual performance related outcomes such as organizational citizenship behaviors (Cropanzano et al., 2003; Ford et al., 2011). Employee wellbeing has been linked to several positive organizational attitudes, including team cohesion, job, and engagement (Bakker, 2015). Promoting wellbeing has the potential of benefiting both the employee and the organization.

## Workplace wellbeing interventions

Traditionally, workplace wellbeing interventions have aimed at reducing stress among employees (Hone et al., 2014). Such interventions have focused on reducing either perceptions of stress, sometimes in combination with increasing employees' ability to cope with stress, which, in turn, has been assumed to increase wellbeing. Organizational interventions are aimed at one or more levels, typically categorized as the primary, secondary, or tertiary levels (Tetrick and Quick, 2011). Primary interventions focus on the organization and aim to reduce or eliminate stressors; secondary interventions focus on changing an individual's perception of, or reaction to a stressor; and tertiary interventions aim at rehabilitating individuals who are suffering from strain in response to stressors (Tetrick and Quick, 2011). Although tertiary stress interventions may provide a short-term improvement in terms of stress reduction for those already suffering from work stress, they do not appear to have a long term effect on individual stress coping, nor on organizational attitudes (Noblet and Lamontagne, 2006). The present study investigates the efficacy of a type of intervention which combines the primary and secondary levels by encouraging individuals to engage in positive activities which promote wellbeing.

## The five ways to wellbeing

The present study investigates the utility of a wellbeing promoting framework called the Five Ways to Wellbeing, implemented through an online tool, the Wellbeing Game. The Five Ways to Wellbeing framework was developed to provide a simple framework to promote mental wellbeing in the general community (Aked et al., 2009). The term “Five Ways” is analogous to a public health campaign in the UK focusing on “Five Fruits and Vegetables a Day” (Aked et al., 2009). The specific “five ways” in the framework were based on scientific evidence on categories of activities that relate to wellbeing. The categories of activities in the framework are *Connect, Be Active, Take Notice, Keep Learning*, and *Give*, and are centered on the importance of social relationships, physical activity, awareness, learning, and giving. These specific actions have been selected for four reasons: They are evidence based, have universal appeal, target the individual, and provide variety, choice, and self-direction in one's daily life, and in the promotion of wellbeing (Aked et al., 2009).

*Connect* involves connecting with others. Developing social connections supports and enriches everyday life as social relationships promote wellbeing and protect against mental ill health (Diener and Seligman, 2002). Feeling close to and valued by other people is a fundamental human need which contributes to functioning well in the world; connecting with people is a key way to wellbeing (Aked et al., 2009).

*Be Active* involves exercising or engaging in some physical activity in an enjoyable way that is suited to individual mobility and fitness levels. Regular physical exercise is linked to a greater sense of wellbeing (Hone et al., 2015). Furthermore, engagement in physical activity increases self-efficacy, perceived ability to cope, and provides a sense of mastery, and thereby promotes wellbeing (Aked et al., 2009).

*Take notice* involves being curious, being be aware of personal emotions and of the world, and reflecting on experiences (Aked et al., 2009). Taking notice is a form of mindfulness, which can be described as being aware of sensations, thoughts, and feelings, and which has been related to wellbeing (Brown and Ryan, 2003).

*Keep Learning* involves trying something new or setting a challenge that one will enjoy achieving (Aked et al., 2009). The continuation of learning throughout life increases self-esteem, and encourages social interaction and a more active life, which in turn has been shown to increase wellbeing (Feinstein and Hammond, 2004). The goal-directed behavior associated with learning, as well as the activity of learning itself, is important for wellbeing (Aked et al., 2009).

*Give* involves doing something nice for someone, a friend, a stranger, or the community. Giving back to the wider community create a sense of connectedness with others, which in turn promotes wellbeing (Aked et al., 2009), by being intrinsically rewarding (Ryan and Deci, 2001).

These Five Ways to Wellbeing are designed to promote positive feedback loops in order to reinforce the engagement in similar and more frequent wellbeing-promoting activities and behaviors (Aked et al., 2009). The Five Ways encourages behaviors which promote both the hedonic and eudaimonic perspective of wellbeing. There is empirical support for a link between the Five Ways activities and flourishing; those who engage in these behaviors are more likely to experience a state of high wellbeing characteristic of flourishing (Hone et al., 2015). While it has not been investigated, the Five Ways categories of activities could be incorporated into the workplace as a way of promoting employee wellbeing (Aked et al., 2009). This could be facilitated by the Wellbeing Game (Mental Health Foundation, 2016), an online tool based on the Five Ways to Wellbeing, which can be used by individuals and organizations to increase their engagement in wellbeing-related activities.

## The wellbeing game

The Wellbeing Game is a free online game designed by The Mental Health Foundation of New Zealand (Mental Health Foundation, 2016) using the Five Ways to Wellbeing as a framework for players to reflect on the positive aspects of their lives. The aim is to make players aware of the wellbeing-enhancing activities they already engage in that to support their own wellbeing and encourage further engagement in such activities. Players log activities which they have taken part in over the course of the day via the online social media platform in the Wellbeing Game. Players then categorize these activities according to one or more of the Five Ways to Wellbeing. The Wellbeing Game draws on aspects of primary psychosocial interventions by developing relationships within teams which promote good functioning and a good social climate. Through the promotion of mindfulness and the building of positive emotions the Wellbeing Game also includes aspects of secondary interventions. Additionally, the Wellbeing Game teaches techniques to address the symptoms of strain, such as physical exercise or seeking social support.

The Wellbeing Game uses gamification to encourage engaging in the Five Ways to Wellbeing through. Gamification enhances a non-game activity with elements typical to a game in order to invoke a game-like experience and thereby increase motivation to partake in the activity (King et al., 2013). The Wellbeing Game applies gamification of the Five Ways to Wellbeing by incorporating a points system, a leaderboard, and rewards in the form of badges in order to increase motivation to engage in the Five Ways, and reinforcing the use of the Five Ways. Points are given based on the length of time for activities logged, and virtual badges are rewarded when specific thresholds are passed.

Gamification can be understood in terms of operant conditioning. The Wellbeing Game uses positive reinforcement to strengthen the likelihood of similar future behaviors occurring (Kapp, 2012). For example, when an activity is logged, points are given, encouraging the player to continue to engage in and logging activities in order to earn more points. Furthermore, when certain point thresholds are passed, badges are given. As the player does not know exactly when the badges will be rewarded, this random reinforcement schedule further increases the desire to engage in the Wellbeing Game.

## The current study

In the present study we investigate whether playing the Wellbeing Game is associated with an increase in players' wellbeing. To investigate this, two studies were conducted. Study 1 was conducted in a student population to test the assumption that the Wellbeing Game works by altering perceptions of visual stimuli. Study 2 was conducted in an organizational context to investigate the effectiveness of the Wellbeing Game in an organizational context. Participants in both studies completed base-line measures of wellbeing, played the Wellbeing Game for a set period of time, and then completed the wellbeing measures again. In Study 1, participants completed a picture categorization task. In Study 2, participants completed an organizational attitudes survey measuring relationships between team members, job engagement, and turnover intentions, as well as a post-intervention survey on perceptions of The Wellbeing Game within the organization. This included perceptions of the Wellbeing Games intrusiveness, alignment with the organizations' values, organizational support for the Wellbeing Game, and its ability to increase connections within the organization.

## Study 1

An evaluation of the 2014 version of the Wellbeing Game indicated that wellbeing increased significantly after playing the Wellbeing Game (Green, 2015). This increase can be explained by two psychological theories: The Broaden and Build Theory (Fredrickson, 2001) and mindfulness theory (Brown and Ryan, 2003).

The Broaden and Build theory states that the function of positive emotions is to broaden a person's thought-action repertoire to build personal resources. A thought-action repertoire refers to a person's set of actions that follow thoughts. Thoughts accompanied by negative emotions are followed by a narrow set of actions. Conversely, positive emotions are followed by a broad set of actions. For instance, joy may be followed by celebrating or sharing with friends (Fredrickson, 2001). The Wellbeing Game encourages players to take part in activities which they enjoy, thereby creating positive emotions. These positive emotions will broaden an individual's mind-set, allowing broader and more creative thinking. In turn, this alters perceptions of potentially stressful situations by allowing a person to frame these situations differently, and increases their resilience in these situations (Fredrickson, 2001). Thus, by being encouraged by the Wellbeing Game to engage in the Five Ways to Wellbeing, players will experience more positive emotions.

The Wellbeing Game also draws on mindfulness techniques. Mindfulness refers to a present-centered attention and awareness, which allows a person to interpret an event as it is, free from personal bias (Brown and Ryan, 2003). This helps a person to function well by increasing engagement in activities of value, thus, it increases wellbeing (Shapiro et al., 2008). When a person is mindful, attention is given to the present moment. The Wellbeing Game supports mindfulness by encouraging players to take notice of the positive experiences in their lives, and by helping players interpret events in a more positive way, wellbeing is facilitated (Aked et al., 2009). It is expected that by increasing the frequency of positive emotions and experiences, the Wellbeing Game will contribute to higher levels of wellbeing.

In Study 1, we investigate the following hypotheses:

*Hypothesis 1a:* Those who have played the Wellbeing Game for 1 week will report an increase in levels of wellbeing as compared to those in a control group.

It is also expected that by playing the Wellbeing Game, the perception of personal resources will increased, which should contribute to fewer perceptions of stress. Therefore, we hypothesize:

*Hypothesis 1b:* Those who have played the Wellbeing Game for 1 week will report a reduction of stress as compared to those in a control group.

By increasing the frequency of positive emotions and decrease in personal biases, the Wellbeing Game should influence players perceiving stimuli in a more positive light. Therefore, we hypothesize:

*Hypothesis 1c:* Those who have played the Wellbeing Game for 1 week will categorize an increased number of stimuli as positive, rather than neutral or negative, compared to those in a control group.

### Study 1 method

#### Participants

The participants were 60 students from the University of Canterbury. The experimental group included 32 participants (24 female and 8 male), and the control group included 28 participants (20 female and 8 male). The mean age across groups was 21.48 (SD = 3.57).

Participants were recruited using a variety of methods. These included advertisements placed around the University of Canterbury as well as in an online research participant forum, through a verbal request to 100 level Psychology laboratory groups, and an email sent to undergraduate Psychology and Commerce students sent by the respective department administrators. During recruitment, potential participants were informed that they would complete two computer based tasks 1 week apart, and that they may also be required to complete a 5 min task once a day during this week. The name or purpose of this task was not disclosed. They were also informed that after completing both stages of the experiment, participants would be compensated with a $10 voucher and placed in the draw to win one of five $130 shopping vouchers.

After signing up, participants were randomly assigned to either the experimental or control condition (the groups were balanced for gender). Participants were then assigned a unique participant number which allowed for each participant to be tracked over time. This number also identified the participant's group membership (experimental vs. control group).

#### Measures and materials

##### Wellbeing

The Short Warwick-Edinburgh Mental Wellbeing Scale (SWEMWBS; Stewart-Brown et al., 2009) was used to measure subjective wellbeing and psychological wellbeing, covering both the hedonic and eudaimonic perspectives of wellbeing. Participants were asked to specify the extent to which they had felt the way described in each of the seven SWEMWBS items over the past 2 weeks. Responses were recorded on a five point Likert type scale (1 = none of the time, 5 = all of the time). A sample item was “I've been feeling optimistic about the future”. The reliability of the scale in this study at Time 1 was Cronbach's α = 0.72 and the reliability at Time 2 was Cronbach's α = 0.78.

##### Stress

A question designed for the purpose of this study was asked in order to assess self-perceived stress. A single item was used in order to increase face validity and to optimize participation by reducing the length of the survey. The question developed was “Stress means a situation in which a person feels tense, restless, nervous or anxious or is unable to sleep at night because his/her mind is troubled all the time. How often do you experience this kind of stress?” Responses were recorded on a five point Likert type scale (1 = none of the time, 5 = all of the time).

##### Stimuli categorization task

The International Affective Picture System (IAPS) is a database of standardized pictures designed for the study of emotion. Each picture has a standardized valence (unpleasant to pleasant), arousal (calm to excited), and dominance (low control to high control) score, ranging from 1 to 9 (Lang et al., 1999). This study used a total of 60 pictures. The picture categories differed in terms of valence ratings. 20 pictures portrayed positive scenes (e.g., family, smiling faces, animals), 20 portrayed neutral scenes (e.g., neutral faces, household objects), and 20 portrayed negative scenes (e.g., sad/angry faces, wreckages, aggressive/attack pictures). The cut-off for each category was predetermined by a classification used in previous research in New Zealand (Flood et al., 2015). Positive pictures were those with a normed valence rating of 6 or above, neutral pictures were those with normed valence ratings above 4 and below 6, and negative pictures were those with a normed valence rating of 4 or less. Arousal and dominance ratings were kept neutral (a rating between 4 and 6) across these categories.

To select these pictures, the IAPS database was ordered on the standardized valence score. Pictures with positive (above 6) or negative (below 4) arousal or dominance ratings were removed. The remaining pictures were split into three categories; those with positive valence ratings, those with neutral valence ratings, and those with negative valence ratings. 20 pictures were selected from each of these three categories. The pictures were then visually inspected and any depicting mutilation or erotica were excluded and replaced with the picture with the most similar valence rating. This was done in order to avoid exposing participants to unnecessary sensitive content. The mean valence ratings of the positive, negative and neutral categories were *M* = 6.64, (*SD* = 1.689), *M* = 4.91 (*SD* = 1.769) *M* = 3.29 (*SD* = 1.678) respectively.

The 60 pictures were randomly ordered with each picture appearing once, but in the same order for all participants.

##### Manipulation check

All participants completed a survey at the end of the study asking whether they had been asked to play, and whether they had played, the Wellbeing Game as part of the study. This was used to ensure that those in the control group had not played the Wellbeing Game.

##### Intervention

Those randomized into the experimental condition, were instructed to play the Wellbeing Game, which was accessed online and free of charge at www.thewellbeinggame.org.nz. To sign up to the Wellbeing Game, players clicked the “Get Started For Free!” button, and they then entered a nickname, email address, password and real name. Gender, age, and ethnicity are then entered and the terms and conditions have to be accepted before the sign up process is complete. Finally, players completed a wellbeing survey (SWEMWBS) before beginning the Wellbeing Game. In this study, participants were instructed to enter their participant number as their real name in order to allow data from the Wellbeing Game to be matched with their experimental data. They were also informed that the wellbeing survey was the same as the survey used in the experiment.

After completing the wellbeing survey, players had the option to join a team, but in the current study, participants played the Wellbeing Game as individuals, therefore they were all instructed to create their own team. In this study, participants created their own team name, indicated that their team is based at a tertiary education facility, and were instructed to call the name of their organization “UCExperiment.” This allowed for easy identification of the players taking part in the experiment, compared to any potential players from the University of Canterbury who were not part of this research. Once sign-up was completed, players were ready to play the Wellbeing Game.

To log an activity players type in a “what did you do” box, indicate how long the activity took, and select the appropriate Ways to Wellbeing (one or more). Once the activity is logged, players are congratulated for completing an activity, and badges are given. In one instance, three badges were received, the Learner Plate, Student of Curiosity, and Ox of Wellbeing. These are given when predetermined landmarks are passed throughout the Wellbeing Game, for instance logging the first activity. These badges are used as rewards for progressing through levels in the Wellbeing Game.

Finally, the Leader Board, Team, and Diary tabs can be viewed to show information on which team is winning, the teams' activities, and personal wellbeing activities, respectively.

#### Procedure

During the first part of the study, participants were individually seated in front of a computer in a room free from distractions at the University of Canterbury, and asked to turn off any personal electronic devices. An information sheet about the experiment was and participants were given the chance to ask questions before signing an informed consent form.

Different information sheets were used for the experimental group and for the control group. These were identical, with the exception for the experimental group being informed that they would take part in an intervention (the name or purpose was not given). Both information sheets contained a small element of deception: Participants were informed that they were taking part in an image categorization experiment, rather than an experiment investigating the efficacy of the Wellbeing Game.

Once this form was signed, the experimenter opened the E-Prime software used to run the experiment and entered the participant number and then instructed the participants to enter their age and gender when prompted. Participants were informed that once they clicked “enter” on screen, the task would begin. The experimenter then left the room and participants began the task which was the same for all participants (experimental and control).

Information onscreen informed the participants that they would complete the survey section of the task, followed by a task involving the categorization of images. Following the last item of the survey, participants were informed that the survey section was finished and that the image categorization section would follow. Participants were then given the following instructions: “*You are asked to categorize each picture into either a positive, negative, or neutral category, dependent on how you interpret that picture. Some of the pictures may prompt emotional experiences, others may seem relatively neutral. Your categorization of each picture should reflect your immediate personal experience, and no more. There are no right or wrong answers, so simply **respond as quickly as you can,** based on your immediate feeling toward the picture.”* This was followed by a description of each of the arrow keys and for which images to use these keys.

Following the completion of three practice images, participants were informed that they were about to begin the actual test, and to remember to respond as quickly as possible. Images were then presented one by one in three blocks of 20 images. Each image remained on screen until a response was recorded. Participants were given a 30 s break in between blocks with a 10 s countdown timer appearing onscreen to signal the end of the break was approaching.

Following the completion of this image categorization task, participants exited the room as instructed to inform the experimenter that the task had been completed. Participants in the control condition were reminded that they would need to return in 1 weeks' time to re-complete the task before receiving their incentive and then dismissed. Participants in the experimental condition re-entered the room with the experimenter and were briefed on the intervention.

Information was provided on how to sign up to play the Wellbeing Game, and how to play it, and the requirement to play every day for the following 7 day period. After the participants were dismissed they were sent an email containing this information, as well as the link to the Wellbeing Game (www.thewellbeinggame.org.nz) and a link to information on the Five Ways to Wellbeing (www.mentalhealth.org.nz/home/ways-to-wellbeing). These participants were sent a reminder text message on days 3, 5, and 7.

Seven days after completing Time 1 testing, participants returned and completed the same survey and image categorization task as at Time 1, the only difference being that all participants (experimental and control groups) completed the task in groups, ranging in size from one to eight participants. Participants were seated in individual cubical workstations in a computer lab at the University of Canterbury. Participants were unable to see the other participants' screens. The same instructions were given as at Time 1 with the addition of the request to not talk, and to wait until all participants had completed the task before leaving. Participants were also asked to complete the manipulation check survey following the completion of the computer based task. Participants were then read a short debrief information sheet which explained the purpose of the experiment more fully. After this they were given the opportunity to ask any question. Finally, the participant incentives were distributed.

Ethics approval for this research was received from the University of Canterbury Human Ethics Committee.

## Study 1 results

This study aimed to investigate whether playing The Wellbeing Game is related to changes in wellbeing, stress, or in how positively participants categorize stimuli. The experiment employed a 2 × 2 mixed repeated measures design. Playing The Wellbeing Game or not playing The Wellbeing Game was the between-subjects variable (experimental vs. control). The repeated measures dependent variables were survey responses, and image category placement.

The responses to the manipulation check survey were checked against each participant's group assignment. No contamination of the control group had occurred.

The assumption of normality was checked by observing the normal Q-Q plots of each of the groups for each of the dependent variables, and all data was deemed sufficiently normal. Assumption of homogeneity of variance between the control and experimental groups was also tested at Time 1 and Time 2, was met for the wellbeing scores and the picture categorization task, as indicated by non-significant Levene's test. For the stress scores, the assumption of homogeneity of variance between the control and experimental groups was met in Time 1 as shown by non-significant Levene's test, *F*_(1, 58)_ = 3.55, *p* = 0.065, but not at Time 2, as shown by a significant Levene's test, *F*_(1, 58)_ = 7.86, *p* = 0.007, log transformed data at Time 1 and Time 2 data was used to resolve this issue. The assumption of homogeneity of variance between the control and experimental groups of this transformed data was met after this transformation, as shown by a non-significant Levene's test at Time 1, *F*_(1, 58)_ = 1.51, *p* = 0.0.224, and at Time 2, *F*_(1, 58)_ = 3.31, *p* = 0.074. The log-transformed data was therefore used in the analysis of the stress scores.

### Hypothesis 1a—changes in wellbeing

Hypothesis 1a suggested that those playing the Wellbeing Game for 1 week would report an increase in wellbeing compared to a control group. A repeated measures ANOVA with a between (experimental vs. control group) and a within subjects variable (wellbeing scores) was used to test for a statistically significant difference between the mean wellbeing scores of the groups after the intervention.

The ANOVA showed that the main effect of group on wellbeing was non-significant, as was the main effect of time on wellbeing. However, in line with Hypothesis 1a, there was a significant interaction effect of game participation and time, *F*_(1, 58)_ = 4.39, *p* < 0.05 η*p*^2^ = 0.07. The experimental group saw an increase in wellbeing at Time 2 (*M*_*Time*1_ = 3.353 [*SD*_*Time*1_ = 0.67] vs. *M*_*Time*2_ = 3.579 [*SD*_*Time*2_ = 0.56) and the control group experienced a very small decrease (*M*_*Time*1_ = 3.414 [*SD*_*Time*1_ = 0.49] vs. *M*_*Time*2_ = 3.352 [*SD*_*Time*2_ = 0.62]).

### Hypothesis 1b—changes in stress

Hypothesis 1b suggested that those playing The Wellbeing Game for 1 week would report a decrease in stress compared to a control group. A repeated measures ANOVA with a between (experimental vs. control group) and a within subjects variable (stress scores) was used to test for a statistically significant difference between the mean stress scores of the groups after the intervention.

The ANOVA showed that the main effect of game participation on stress was non-significant, as was the main effect of time on stress. There was also no significant interaction effect between game participation and time point on stress, indicating that stress scores did not change as a function of the Wellbeing Game, and Hypothesis 1b was not supported.

### Hypothesis 1c—changes in picture categorization

This analysis tested Hypothesis 1c, suggesting that those who played the Wellbeing Game would place more pictures in a more positive category than those who did not play the Wellbeing Game. To place a picture in a more positive category would entail either a change from a negative to a neutral categorization; a change from a neutral to a positive categorization; or a change from a negative to a positive categorization.

The categorization task responses were first checked for errors. A minimum of 300 ms is required to process and response to visual stimuli (Greenwald et al., 2003). Therefore, responses faster than 300 ms were identified as failures to inhibit a response and removed from further analysis. This resulted in the removal of a total of 17 responses (0.21%) from 11 different participants (5 experimental and 6 control).

Data was inspected to ensure that participants had used the correct response keys. To do so, a marking variable was created for each participant which showed whether each picture had been correctly categorized according to the standardized valence score determined by the International Affective Picture Systems (IAPS; Lang et al., 1999). Participants who had incorrectly categorized 50% or more pictures at either Time 1 or Time 2 were excluded from further analysis, based on the assumption that if the positive and negative response keys were used the wrong way around, a participant who would have otherwise categorized every picture correctly would unintentionally categorize 67% of pictures incorrectly (the 20 neutral pictures would be unaffected). However, when the correct keys are used, the most likely source of error is miscategorizing a neutral picture or a picture that was bordering on the neutral category (negative rating between 3.5 and 4, positive rating between 6 and 6.5). With the pictures used, this accounts for only 39% of stimuli. Therefore, the cut off for exclusion was set at 50% to allow for some variation in valence perception but to exclude data which was not valid. Also, it was determined that this error should not be corrected for by reversing responses (i.e., replace “positive” responses for “negative” responses and vice versa), to avoid introducing further error into the data, These participants were therefore deleted from further analysis (Time 1 and Time 2 data). This resulted in the removal of 10 participants (5 experimental and 5 control).

A Rating Index was calculated using the remaining data. Each response was assigned a value which reflected the participant's category placement of each stimulus picture. A negative response was assigned the value −1, a neutral response was assigned the value 0, and a positive response was assigned the value 1. The mean categorization rating was then calculated for each participant which reflected the proportion of images placed in each category. The categorization index had a range of −1 to 1, with −1 meaning all images were categorized as negative, and 1 meaning all images were classed as positive.

A repeated measures ANOVA with a between (experimental vs. control group) and a within subjects variable (picture categorization) was used to test for a significant difference between picture categorization between the experimental and control groups after playing the Wellbeing Game.

The results showed that there were no significant main effects of group or time, and there was no significant interaction between group and time point, *F*_(1, 48)_ = 0.33, *p* = 0.569, η*p*^2^ = 0.07. However, when inspecting the means for the different groups, there was an indication that those in the experimental group to categorize pictures more positively in Time 2 than in Time 1 (*M*_*Time*1_ = −0.156 [*SD*_*Time*1_ = 0.167], *M*_*Time*2_ = −0.123 [*SD*_*Time*2_ = 0.164]) and for those in the control group to categorize stimuli more negatively (*M*_*Time*1_ = −0.185 [*SD*_*Time*1_ = 0.179], *M*_*Time*2_ = −0.196 [*SD*_*Time*2_ = 0.181]).

The mean number of pictures placed into each of the three categories was then inspected. This showed a consistent trend. At Time 2, the experimental group placed fewer pictures in the negative group and more in the positive and neutral categories, and the control group placed more pictures in the negative category, and fewer in the positive and neutral categories (Table 1). However, a repeated Multivariate Analysis of Variance (MANOVA) where the repeated measured variables were positive category placement, neutral category placement, and negative category placement and the between subject variable was game participation (experimental vs. control) showed that these differences were all non-significant. Thus, Hypothesis 1c was partially supported.

**Table 1:** **Means and standard deviations of the number of pictures placed in each category across T1 and T2**.

***Stimulus Valence***	**Experimental Group**				**Control Group**				***F***	***p***
	**T1**		**T2**		**T1**		**T2**			
	***M***	***SD***	***M***	***SD***	***M***	***SD***	***M***	***SD***		
Positive	17.15	6.22	17.20	6.11	16.55	6.50	16.25	5.570	0.072	0.789
Neutral	16.69	6.66	17.68	7.01	16.00	5.81	15.40	10.01	0.015	0.902
Negative	26.15	6.20	24.96	6.28	27.35	6.23	28.20	8.051	0.013	0.911

*F and p-values refer to the interaction between group and time on category placement*.

### Study 1 discussion

Study 1 investigated whether students would report higher levels of wellbeing and lower levels of stress after playing the Wellbeing Game compared to a group of students not playing the Wellbeing Game. Study 1 also investigated whether students would change their likelihood to perceive stimuli as positive after playing the Wellbeing Game, as indicated by a picture categorization task. Hypothesis 1a was supported; students playing the Wellbeing Game reported an increase in wellbeing. However, Hypothesis 1b was not supported; those playing the Wellbeing Game did not report a decrease in stress. Hypothesis 1c, suggesting that student players would place more images in a more positive category after playing the Wellbeing Game was also not supported. However, there was an indication that those in experimental condition placed more images in the positive category and that those in the control condition placed more images in the negative category after the intervention. The finding that playing the Wellbeing Game may be related to increased wellbeing is consistent with the evaluation of the 2014 version of the Wellbeing Game which found a significant increase in self-reported wellbeing (Green, 2015). There was a major methodological difference between these studies. In the current study, participants played the Wellbeing Game individually, whereas in the evaluation by Green (2015), a significant portion of participants played the Wellbeing Game in a team. Green (2015) found that there was no difference in the change in wellbeing for those who played the Wellbeing Game in a team and those who did not. The team aspect of the Wellbeing Game may not be a central aspect contributing changes in wellbeing.

The positive change in wellbeing is also in line with research linking engagement with the Five Ways to Wellbeing to higher levels of flourishing (Hone et al., 2015). However, that previous research (Hone et al., 2015) used a cross sectional design, whereas the longitudinal (albeit with a short timeframe) design of the current study indicates that engaging with the Five Ways to Wellbeing through the Wellbeing Game may potentially effect changes in wellbeing over time.

While wellbeing increased for those in the experimental condition, it actually decreased for those in the control condition who did not play the Wellbeing Game. The intervention took place during the last weeks of the University year, and many participants had final tests and assignments due during the Time 2 survey. Therefore, it may be reasonable that wellbeing would decrease at this point in time among those who did not engage in any wellbeing-promoting activities. However, in the experimental group, who played the Wellbeing Game, it seems that wellbeing was actually improved, despite the time of year. This may be an indication that this intervention could be promoted to at-risk-members of society, for example students during exam time, to prevent a reduction in wellbeing.

The heavy workload experienced by participants due to end of year tests and assignments may explain why Hypothesis 1b was not supported; there was no change in stress after playing the Wellbeing Game. This finding aligns with research that shows that wellbeing and stress exist on separate, although overlapping spectrums (Keyes, 2005).

After playing the Wellbeing Game, players did not place *significantly* more pictures in a more positive category compared to those who did not play the Wellbeing Game. However, on visual inspection those in the experimental condition placed slightly fewer pictures in the negative category after having played the Wellbeing Game, and those in the control condition to place slightly more pictures in the negative category at Time 2. This pattern of change in picture categorization may be interpreted in light of the significant patterns in the changes of wellbeing. Wellbeing increased for those who played the Wellbeing Game and decreased for those who did not. While this result needs further investigation in a different, larger sample, it may be an initial indication that changes in how stimuli are perceived do relate to the changes in wellbeing.

It is possible that the small changes in picture categorization patters, would have been statistically significance if more time was spent playing the Wellbeing Game, as the mechanisms assumed to link the Wellbeing Game to increases in wellbeing may take time to have any observable effect. Changes in positive emotions and behaviors works in two stages. First, positive emotions result in a broader thought-action repertoire, then as a result, personal resources are built through engagement in a wider variety of activities. Small incremental changes in the availability of personal resources overtime eventually result in a large effect (Fredrickson and Joiner, 2002). Based on this, it is reasonable to expect more of an increase in wellbeing than the perception of the valence of the stimuli in the categorization task, since a key aspect of wellbeing is experiencing positive emotions.

Additionally, mindfulness is a state which takes much practice to achieve. Although many workplace mindfulness training programs take only a few hours to complete, daily practice is required in order to become a mindful person, with many people viewing mindfulness as a lifelong pursuit (Brown and Ryan, 2003). Therefore, it is likely that more exposure to the Wellbeing Game may be needed before any increase in the perception of positive stimuli would occur.

## Study 2

Study 1 investigated whether students playing the Wellbeing Game would report increases in wellbeing, decreases in stress, and increases in the perception of stimuli as positive. Since Study 1 utilized a student sample, it is important to investigate whether the Wellbeing Game can be used in an organizational setting as well. Given that employee wellbeing is likely influenced to a large extent by activities in the workplace, implementing the Five Ways to Wellbeing in the workplace setting by encouraging employees to play the Wellbeing Game may be related to positive outcomes. In Study 1 participants only played the Wellbeing Game for a week, which may be too short of a time period to affect any large changes. However, Study 1 showed that participants playing the Wellbeing Game was reported positive outcomes, even over the short time period, and it is expected that playing the Wellbeing Game should be related to a positive change in wellbeing among employees playing the Wellbeing Game at work.

### Study 2 hypotheses

Study 2 tests the following hypotheses:

*Hypothesis 2a:* Employees playing the Wellbeing Game will report higher levels of wellbeing after playing the Wellbeing Game for a month.

It is expected that by playing the Wellbeing Game, perceptions of availability of personal resources should be increased which should decrease perceptions of stress.

*Hypothesis 2b:* Employees playing the Wellbeing Game for 1 month will report lower levels of stress.

Finally, engaging in the Five Ways to Wellbeing is linked to flourishing (Hone et al., 2015), which is relates to more positive organizational attitudes such as low turnover intentions and high job engagement and more positive relationships between team members.

*Hypothesis 2c*: Employees playing the Wellbeing Game for 1 month will report a positive change in employee attitudes toward the organization, including lower turnover intentions, higher job engagement and more positive evaluations of relationships between team members.

### Study 2 method

#### Participants

The participants were 52 employees from a large financial organization in New Zealand. This organization was approached in collaboration with the Mental Health Foundation of New Zealand who was responsible for contacting the organization's Wellbeing Champion (the person in charge of promoting wellbeing within the organization). Participants volunteered to participate in response to a request from the Wellbeing Champion in the organization. 157 employees completed the pre-game survey, and 54 employees completed the intervention and the post-game survey.

#### Measures

##### Wellbeing

The Short Warwick-Edinburgh Mental Wellbeing Scale (SWEMWBS; Stewart-Brown et al., 2009). The reliability of the scale in this study at Time 1 was Cronbach's α = 0.86 and the reliability at Time 2 was also Cronbach's α = 0.86

##### Stress

Study 2 used the same question as the one used in Study 1. This single-item measure was in this study to ensure comparability across studies, and that as little of the organization's time was used as possible.

##### Organizational attitudes

Three items were used to measure three organizational attitudes which were perceived relationships between team members, turnover intentions, and job engagement. Single-item measures were used in order to minimize the time commitment in order to increase the likelihood of participants completing the survey. To assess perceived relationships, the following question was used: “There are good relationships between team members” (Senior and Swailes, 2007); for the turnover intentions: “I am happy to stay with this organization for the next 2 years” (designed for this study) and for job engagement: “I am highly engaged in this job” (Saks, 2006). Responses to these items were recorded on a five point Likert type scale (1 = strongly disagree, 5 = strongly agree).

##### Perceptions of the wellbeing game

Four additional questions were included in the post-game survey at the conclusion of the Wellbeing Game period, in order to assess players' perceptions of how well they thought the Wellbeing Game was integrated in their everyday work, and their general experience of the Wellbeing Game. These were “How does the Wellbeing Game relate to regular work activities—is the Wellbeing Game intrusive?” (1 = intrusive, 5 = integrative). “Does the Wellbeing Game make sense given what your organization stands for? (mission, vision, values)” (1 = not at all, 5 = to a large extent). “Do people support the Wellbeing Game in your organization? (1 = not at all, 5 = to a large extent). “This Game has enabled me to connect more with others” (1 = strongly disagree, 5 = strongly agree). These questions were designed in collaboration with the Mental Health Foundation of New Zealand for the purpose of this study.

#### Intervention

The Wellbeing Game was the same as in Study 1, except for that in this study participants played the Wellbeing Game for 4 weeks, and in teams, and were encouraged by the organization to engage with the Wellbeing Game. Participants were instructed to join the team assigned to them by the organization's Wellbeing Champion.

#### Procedure

At the beginning of the month during which the Wellbeing Game was played, the organization's wellbeing champion, who was in contact with the Mental Health Foundation, emailed participants the Wellbeing Game set up information. When setting up an account, participants completed the wellbeing, stress and organizational attitudes survey. Participants then played the Wellbeing Game for a period of 1 month. At the completion of the month, participants were sent a post-game email from the Wellbeing Champion. This email contained information on the winners of the Wellbeing Game, the number of teams that played and total hours logged. A request to complete the post-game survey, containing the same questions as the pre-game survey with the addition of the four post-game questions was included in this email. Participants then completed the post-game survey which included the same measures as the pre-game survey.

### Study 2 results

The study tested whether employees playing the Wellbeing Game within an organization for a 1 month period would report higher levels of wellbeing, lower levels of stress, as well as more positive organizational attitudes.

#### Hypothesis 2a—changes in employee wellbeing

Hypothesis 2a suggested that after playing the Wellbeing Game, employees would report higher levels of wellbeing. The results indicate an increase in means, suggesting that wellbeing increased after the Wellbeing Game was played for the month. The mean wellbeing at Time 1 was 3.75 (*SD* = 0.48) and the mean wellbeing at Time 2 was 3.84 (*SD* = 0.51). However, a paired samples *t-*test showed that when using the traditional cut-off of 0.05 for significance [*t*_(51)_ = −1.642, *p* = 0.11], this was not a statistically significant difference. However, the probability that the results represent there being no difference at all is still quite small (*p* = 0.11), suggesting that there is some difference over time in wellbeing scores.

**Figure 1:**
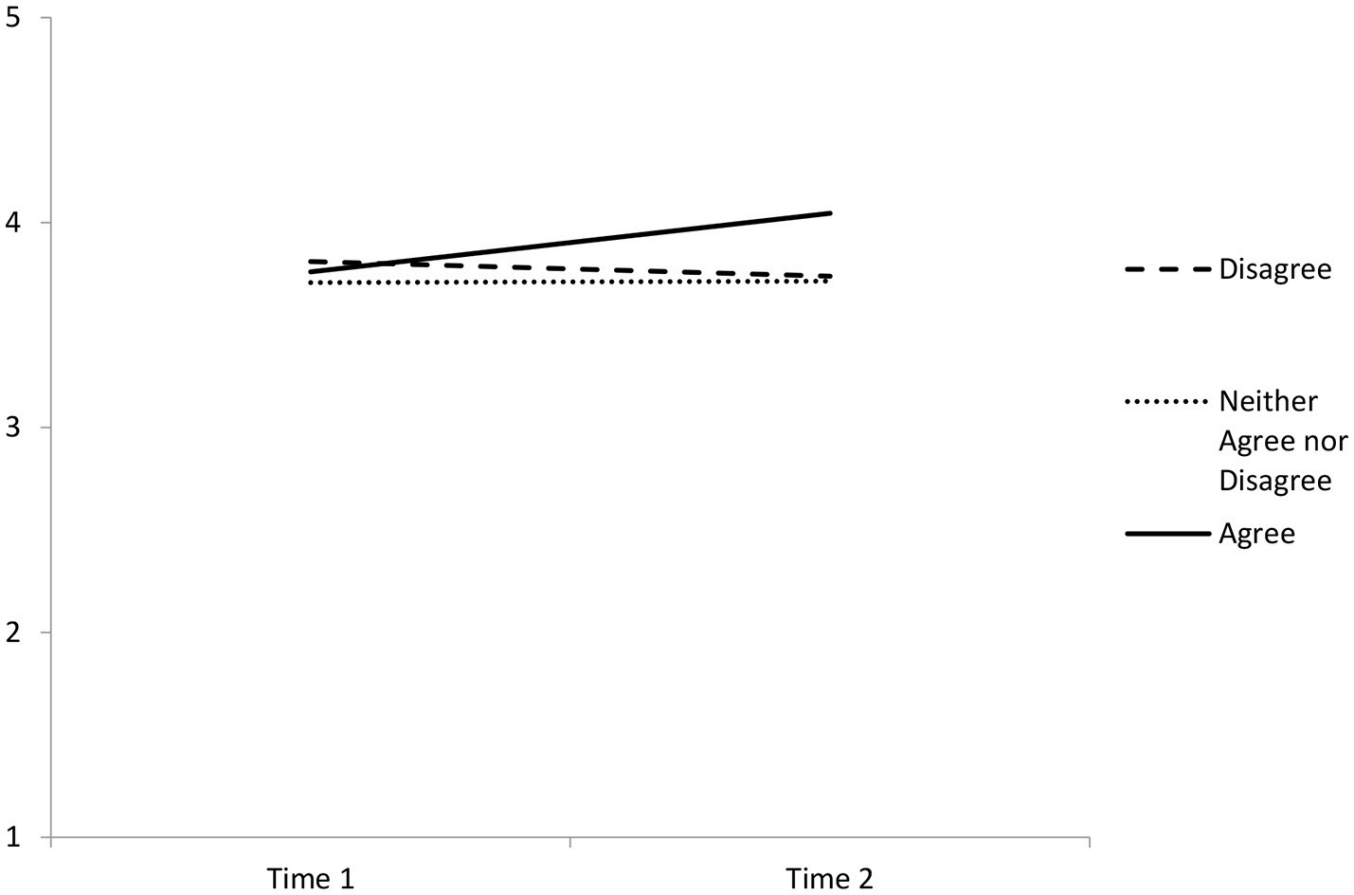
**Wellbeing scores at Time 1 and Time 2 when the degree to which the Wellbeing Game was perceived to increase connections with others was controlled for**.

Participants had commented to the organization and in the survey that they perceived the Wellbeing Game to be effective. To explore these comments empirically, four repeated measures Analyses of Covariance (ANCOVAs) were conducted. The covariates included in the post-game survey were included to shed some light on under what conditions participants found the Wellbeing Game effective. The repeated measures variable was wellbeing at Time 1 and at Time 2, and the covariates used were the four Post Survey question. Given the small number of participants, the five possible groups that could be created using the survey responses (1—5 Likert type scale) were collapsed into three groups (1 and 2 were combined to create a Disagree category; 4 and 5 were combined to create an Agree category) to increase the number of participants in each group. There was no significant difference between the groups when controlling for either of the Intrusive, Makes Sense, or Support for Game questions. However, when controlling for the degree to which employees felt the Wellbeing Game had enabled them to connect more with others (Connect), the increase in wellbeing was significant [*F*_(1, 49)_ = 4.212, *p* = 0.021, η*p*^2^ = 0.147]. When plotted, this shows that those who felt the Wellbeing Game helped them to connect more with others reported an increase in wellbeing (Connect responses of 4 or 5 [Agree]), but that those who did not think the Wellbeing Game helped them connect reported stable, or a slight decrease in, wellbeing levels (see Figure 1).

#### Hypothesis 2b—changes in employee stress

Hypothesis 2b suggested that after playing the Wellbeing Game, employees would report lower levels of stress levels. The mean stress score at Time 1 was 2.92 (*SD* = 0.74) and the mean stress at Time 2 was 2.75 (*SD* = 0.76), and a paired samples *t-*test indicated that stress levels were significantly lower at Time 2, [*t*_(51)_ = −2.021, *p* = 0.049], providing support for Hypothesis 2b.

#### Hypothesis 2c—organizational attitudes

Hypothesis 2c suggested that playing the Wellbeing Game in an organization would influence employees' perceptions of three organizational attitudes. These were perceptions of the quality of the relationships between team members, turnover intentions, and job engagement. To test this hypothesis, three repeated measures analyses of variance (ANOVAs) were conducted. No significant changes were found in any of the three organizational attitudes.

As a follow-up test based on research suggesting that organizational attitudes may improve when wellbeing is high (Hone et al., 2015), three repeated measures ANCOVAs were run using the Time 2 wellbeing measure as a covariate and the three organizational attitudinal variables as the dependent variables. However, no significant differences in any of these organizational attitudes were found. Hypothesis 2c was not supported.

### Study 2 discussion

Study 2 investigated whether those playing the Wellbeing Game in an organization would report increased wellbeing, higher job engagement, decrease turnover intentions, and more positive relationships between team members. Three hypotheses were investigated. The support for Hypothesis 2a, suggesting that employees would report higher wellbeing after playing the Wellbeing Game, was mixed. Wellbeing was significantly higher after playing the Wellbeing Game, but only for those who felt the Wellbeing Game had helped them make more connections with colleagues. This suggests that the extent to which the Wellbeing Game facilitates social connections is important for its efficacy. Positive social connections has been related to higher levels of wellbeing (Diener and Seligman, 2002), which may explain why the increase in wellbeing was only observed for those who also felt that social connections were also improved after playing the Wellbeing Game.

Hypothesis 2b was supported; after playing the Wellbeing Game employees reported lower levels of stress. This finding is in line with previous research which suggests that primary (interventions targets at the organization) and secondary (interventions targets at the individual) stress management interventions are effective at reducing stress, particularly in the short term (Noblet and Lamontagne, 2006). The Wellbeing Game is a combination of primary and secondary interventions, which both promote proactive behaviors and assists in reframing perception of stressors, thus contributing to lower stress. The results of Study 2 suggests that the Wellbeing Game may have some efficacy in contributing to lower levels of employee stress.

Finally, there was no change in any of the organizational attitudes after playing the Wellbeing Game; Hypothesis 2c was not supported. Wellbeing may be a prerequisite for more positive work-related attitudes and behaviors (cf. Cropanzano et al., 2003; Ford et al., 2011), such as higher job engagement, fewer turnover intentions, and more positive relationships between team members. Since, the increase in wellbeing was not substantial, and perhaps this explains why there was no improvement in the work-related outcomes.

In a comprehensive review of resilience interventions, the only study which found no effect used an online intervention (Robertson et al., 2015), which is explained by the limited engagement in the online platform. Participants in Study 1 (the student sample) were informed that they had to play the Wellbeing Game daily in order to receive their incentive, making it more likely that they would engage in the Wellbeing Game. However, in Study 2 there was no tangible incentive for participants to play the Wellbeing Game. Therefore, there may have been less engagement in the Wellbeing Game in Study 2 compared to Study 1, partially explaining that wellbeing was not significantly higher after playing the Wellbeing Game in Study 2.

It is possible that the competitive team aspect of the Wellbeing Game actually contributed to a lack of increase in the perception of the quality of the relationships between colleagues. The team aspect of the Wellbeing Game is expected to help facilitate social connections, and wellbeing only increased among those Study 2 who felt that social connections had been improved. Not all players felt that social connections had been improved. Furthermore, comments from players indicated some dissatisfaction with the competitive element of the Wellbeing Game, and perhaps the competitive aspect impeded social connections for some. Green (2015) found that there was no significant difference in the increase in wellbeing between those who played in a team and those who did not, and further investigation should be conducted to ascertain the function of the team aspect of the Wellbeing Game.

## General discussion

Combining the results from the two studies, the findings show that those playing the Wellbeing Game reported less stress and somewhat higher wellbeing after playing the Wellbeing Game. By encouraging the use of activities within the Five Ways to Wellbeing framework, it appears that the Wellbeing Game contributes to an increase in the frequency of positive emotions. These positive emotions encourage a person the engagement in varied, novel, and exploratory thoughts and actions (Fredrickson, 2001), which build personal resources and resilience to stress. The positive emotions associated with engaging with the Wellbeing Game should motivate players to continue to pursue positive activities which can be linked to The Five Ways, creating a cyclic relationship where playing the Wellbeing Game contributes in positive emotions which lead to engagement in more positive activities, which in turn lead to more positive thoughts. Furthermore, as the Wellbeing Game is expected mindful awareness, which is related to positive emotions, and which in turn should encourage more positive activities. This builds personal resources, including resilience to stress and wellbeing (Fredrickson, 2001). The current study presents preliminary evidence for this type of positive gain spiral (Fredrickson and Joiner, 2002), but more longitudinal research is needed to explore this further.

## Strengths and limitations

The study may have a few limitations which should be taken into account when interpreting the results. In Study 1, the same picture stimuli were used in Time 1 and Time 2 and these were presented in the same order. Thus, participants may have remembered the pictures, making a change in category placement less likely. However, changing the order of the picture presentation could have influenced the category placement. If a very negative photo was presented before a neutral photo, the negative feelings induced by the negative photo may have had an effect on the perception of the following images. Therefore, keeping the same order was the best option as this would not introduce a new, potentially confounding variable.

Additionally, the experiment in Study 1 was not carried out in a lab which meant that the independent variable could not be isolated from other potential confounding variables. Therefore, other events occurring in the participants' lives may have affected the results. This non-lab setting is both a strength and a potential limitation. Although this lack of isolation means that not all extraneous variables could be controlled for, it adds an element of reality to the research. In practice the Wellbeing Game is not used in an isolated environment. Therefore, the fact that the results show that the Wellbeing Game is effective in a real world environment strengthens the utility of this research.

In Study 2, no control group was used, making any comparison between those who played the Wellbeing Game in an organization and those who did not play could not be made. However, given the organizational context in which the Wellbeing Game was played, a comparable control group free from contamination would have been extremely difficult to achieve. The Wellbeing Game is a resource that is provided free to the public, and it is likely that other members of the organization may have been made aware of the Wellbeing Game, and there would be no way to stop these people from playing themselves. While the use of a true control group may have been impractical in this study, it is possible that future research can use other control mechanisms, such as the use of a waitlist or phased intervention.

The sample size used was a limitation in both studies. The small number of participants means that the power of both studies is limited. As only 60 and 52 participants were used in Study 1 and Study 2 respectively, there may not have been enough power to identify any differences between groups. This means that the non-significant findings may actually be due to a lack of power rather than a true absence of a difference. However, this is the first quasi-experimental evaluation of the Wellbeing Game, and future research should explore the findings of this study using a larger sample size.

Both studies utilized single-item measures. While a single-item may be less ideal than a multi-item scale, a single item was preferred as it decreased survey length. Also, the item was specifically developed for the current study in order to clarify to the meaning of the word “stress.” Many of the existing stress measures ask about stress indirectly and in the present study it was important to assess participants' own view of their stress experiences. Furthermore, single item measures have been found to be reliable when measuring self-reported stress (Fisher et al., 2016), and in both studies it was of practical importance to make the survey as short as possible to ensure organizational participation and facilitate survey completion rates.

Both studies utilized a longitudinal design, measuring the dependent variables both before and after an interventions. The time frames used may have influenced the magnitude of the change between the two time points. Longitudinal research should ensure that the study time frames correspond with the underlying mechanism of the change in order to avoid insufficient time for a change to occur, or to not allow too much time before re-testing that any effects have dissipated (Taris and Kompier, 2014). Although the underlying mechanisms contributing to how the Wellbeing Game works (based on mindfulness and Broaden and Build theories) take time to have an effect, a 1 week period was chosen in Study 1 based on the assumption students were unlikely to commit to playing the Wellbeing Game for longer than a week. While this is a short timeframe, previous research indicates that playing the Wellbeing Game at least three times resulted in an increase in wellbeing (Green, 2015), and this supported that 1 week could be sufficient. However, there was no indication in the previous research as to over how long of a period these three game-plays occurred; it is possible that they occurred over longer than 1 week.

Study 2 used a 4-week timeframe, which is consistent with the timeframe used in the Green (2015) study, but in a much smaller sample, which may explain why the results of Study 2 differed from those of Green (2015), and Study 1. However, there was indication that wellbeing was higher among some of those playing the Wellbeing Game in Study 2, and future research should investigate the effect of the Wellbeing Game on perception of stimuli over a longer period of time and in larger samples.

## Conclusion

This research presented two studies of the evaluation of the Wellbeing Game in a student context. This research is also the presents first time that the Wellbeing Game was used and evaluated in an organizational setting. The results showed that after playing the Wellbeing Game students reported higher wellbeing, and employees reported lower stress levels. Additionally, the results showed that the Wellbeing Game has was related to lower stress and higher wellbeing among employees when social connections are improved as a function of the Wellbeing Game. The finding that the Wellbeing Game is more effective in an organization when the degree to which employees feel it helped strengthen connections with those around them is important, highlighting the importance of social support in organizations. Organizations should ensure that wellbeing interventions work to increase the quality of workplace relationships. Finally, this research is the first to investigate the mechanisms behind how the Wellbeing Game works to increase wellbeing by using a quasi-experimental setting. Future studies can build on the design of the current study to further explore the mechanisms behind the Wellbeing Game. Overall, the present study indicates that the Wellbeing Game is a cost-effective tool of engaging students and employees in positive activities with the potential of improving wellbeing in their everyday lives.

## Author contributions

As the first author AK was involved in all steps of the process, and was the primary writer of the text. As the primary supervisor of AK's Master's thesis, KN has been involved in the design, data collection and analysis, as well as the write up of the text. KN, along with the two co-authors, have also been involved in adapting the manuscript from the original thesis. As associate supervisor SM has been involved in the design and analysis of Study 1, as well as data collection and analysis of Study 2, and has contributed to the write-up. As associate supervisor JK has been involved in the design and analysis of Study 2, as well as data collection of Study 2, and has contributed to the write-up.

### Conflict of interest statement

The authors declare that the research was conducted in the absence of any commercial or financial relationships that could be construed as a potential conflict of interest.
